# The Role of the Lung’s Microbiome in the Pathogenesis and Progression of Idiopathic Pulmonary Fibrosis

**DOI:** 10.3390/ijms20225618

**Published:** 2019-11-10

**Authors:** Paolo Spagnolo, Philip L. Molyneaux, Nicol Bernardinello, Elisabetta Cocconcelli, Davide Biondini, Federico Fracasso, Mariaenrica Tiné, Marina Saetta, Toby M. Maher, Elisabetta Balestro

**Affiliations:** 1 Respiratory Disease Unit, Department of Cardiac Thoracic, Vascular Sciences and Public Health, University of Padova, Via Giustiniani 2, 35128 Paolo, Italy; ecocconcelli@icloud.com (E.C.); dav.biondini@icloud.com (D.B.); federico.fracasso@gmail.com (F.F.); mariaenrica.tine@gmail.com (M.T.); marina.saetta@unipd.it (M.S.); elisabetta_balestro@hotmail.com (E.B.); 2 NIHR Respiratory Clinical Research Facility, Royal Brompton Hospital, London SW3 6LR, UK; p.molyneaux@imperial.ac.uk (P.L.M.); t.maher@rbht.nhs.uk (T.M.M.); 3 National Heart and Lung Institute, Imperial College, Sir Alexander Fleming Building, London SW7 2AZ, UK; 4 Respiratory Disease and Lung Function Unit, Department of Medicine and Surgery, University of Parma, 43126 Parma, Italy; nicol.bernardinello@studenti.unipr.it

**Keywords:** idiopathic pulmonary fibrosis, interstitial lung disease, microbiome, pathogenesis, acute exacerbation, infection

## Abstract

Idiopathic pulmonary fibrosis (IPF) is a chronic, progressive, fibrosing interstitial lung disease that commonly affects older adults and is associated with the histopathological and/or radiological patterns of usual interstitial pneumonia (UIP). Despite significant advances in our understanding of disease pathobiology and natural history, what causes IPF remains unknown. A potential role for infection in the disease’s pathogenesis and progression or as a trigger of acute exacerbation has long been postulated, but initial studies based on traditional culture methods have yielded inconsistent results. The recent application to IPF of culture-independent techniques for microbiological analysis has revealed previously unappreciated alterations of the lung microbiome, as well as an increased bacterial burden in the bronchoalveolar lavage (BAL) of IPF patients, although correlation does not necessarily entail causation. In addition, the lung microbiome remains only partially characterized and further research should investigate organisms other than bacteria and viruses, including fungi. The clarification of the role of the microbiome in the pathogenesis and progression of IPF may potentially allow its manipulation, providing an opportunity for targeted therapeutic intervention.

## 1. Introduction

The term “microbiome” refers to the “ecological community of commensal, symbiotic and pathogenic organisms that share our body space,” [1] as well as the complex interactions of these microbes with the host. The gastrointestinal microbiome, composed of more than 100 trillion microorganisms, is the most extensively studied [2]; conversely, the epithelial surface of the lower respiratory tract, one of the least populated surfaces of the human body, has historically been described as sterile. This incorrectly held doctrine arose primarily because of the challenge of directly sampling the lower airways and the limitations of bacterial culture, which prevented isolation and identification of microbes. The transition from culture-dependent to culture-independent methodologies has revealed the complex and dynamic community of microbes harboured by the respiratory tract. Nowadays, high-throughput DNA sequencing technologies enable rapid identification of complex bacterial communities (including organisms that cannot be cultured) based on sequence similarities in highly conserved genes, such as the 16S ribosomal RNA gene (16S rRNA). As a result, researchers have started to examine the lung microbiome in healthy subjects as well as individuals affected by chronic respiratory diseases, such as chronic obstructive pulmonary disease (COPD), bronchiectasis, cystic fibrosis, asthma and interstitial lung disease (ILD). In so doing, various and complex populations of bacteria, fungi and viruses have been identified [3]. 

Idiopathic pulmonary fibrosis (IPF) is a chronic, progressive and ultimately fatal fibrotic ILD of unknown cause that primarily affects older adults [4]. While the mechanisms underlying IPF are incompletely understood, the disease is believed to result from abnormal wound-healing mechanisms following repetitive alveolar microinjury, with smoking, microaspiration of gastric content and infection, among others, representing plausible putative triggers of the fibrotic response [5]. Specifically, infectious agents, including viruses and bacteria, can induce alveolar epithelial cell damage and apoptosis, and modulate the host response to injury [6]. In addition, mutations in the *MUC5B* gene, which encodes a mucin required for normal macrophage function and effective muco-ciliary clearance of bacteria in mice [7], is associated with an increased risk of developing both familiar and sporadic IPF [8], suggesting that bacteria may act as a cofactor in fibrosis initiation in genetically predisposed individuals. Infection may also play a role in disease progression in patients with IPF, in whom active infection carries a high morbidity and mortality, [9] whereas immunosuppression (e.g., combination prednisone, azathioprine and *N*-acetylcysteine) increases the risk of death and hospitalizations [10]. Notably, the utility of corticosteroids in IPF, particularly in the acute phase of the disease, had been questioned well before the PANTHER trial [11,12,13].

In this review, we summarize and critically discuss current knowledge about the interplay between the lung microbiome and IPF, with emphasis on its potential role in disease development, progression and acute exacerbation.

## 2. Infection and Lung Microbiome in Idiopathic Pulmonary Fibrosis (IPF)

### 2.1. Viral Infection

Historically, studies investigating the role of chronic infection as either an etiologic agent or co-factor in the development of IPF have focused primarily on viruses. Ueda and colleagues evaluated the prevalence of serum antibodies to hepatitis C virus (HCV) in patients with IPF (*n* = 66) and found that it was significantly higher compared to age/gender-matched controls (*n* = 9464; 28.8% versus 3.66%, respectively) [14]. In addition, in a cohort of 6150 patients infected with HCV, Arase and colleagues observed a 10-year and 20-year cumulative incidence of IPF of 0.3% and 0.9%, respectively, compared to no cases of IPF in a control group of 2050 patients with hepatitis B virus (HBV) (*p* = 0.02) [15]. The risk of developing IPF was particularly high among heavy smokers, individuals older than 55 years and patients with liver cirrhosis. However, the HCV association with IPF has not been consistently observed [16]. The human herpes viruses (HHVs), a large family of DNA viruses that includes herpes simplex virus type 1 (HSV-1), Epstein-Barr virus (EBV), cytomegalovirus (CMV) and HHV-7 and HHV-8, have received the most attention as causative factors in IPF, mainly because of their ability to cause lifelong latent infection in the alveolar epithelium and to reactivate in older individuals [17]. In the first study to suggest an association between HHV and IPF, 10 out of 13 patients with IPF had raised serum antibodies to EBV compared to none of 12 diseased controls with non-IPF ILD, whereas serum antibodies to HSV and CMV were within the normal ranges in all patients [18]. A number of studies have reported an increased frequency of EBV in lung biopsy and bronchoalveolar lavage (BAL) samples from patients with IPF compared to controls [19,20,21,22]. Folcik and colleagues found DNA from herpes virus saimiri, a pathogen of squirrel monkeys that infects up to 7% of humans, in the regenerating epithelial cells of 21/21 IPF biopsies compared to none of the control lung epithelial cells [23]. Notably, the sequence of the herpesvirus saimiri gene extracted from an IPF sample matched 100% with the published viral sequence, consistent with IPF representing herpesvirus saimiri-induced pulmonary fibrosis [23]. HHV-infected epithelial cells from patients with IPF have evidence of endoplasmic reticulum stress and apoptosis, suggesting a mechanistic link between viral infection and the development of IPF [24]. More recently, Kropski and co-workers found increased herpes virus DNA in cell-free BAL, along with evidence of herpes virus antigen expression in alveolar epithelial cells of asymptomatic relatives of patients with familial IPF, suggesting that the alveolar epithelium of individuals at risk for IPF may be infected with herpes viruses well before the disease becomes clinically evident [25]. However, to a large extent, the data surrounding the role of viral infection in the pathogenesis of ILD remain conflicting and inconclusive.

### 2.2. Lung Microbiome

Initial studies investigating the role of the lung microbiome as a trigger or co-factor in the development and progression of IPF were based on culture-dependent techniques. Richter et al. investigated bacterial colonization of the lower airways in patients with Wegener granulomatosis (WG) (*n* = 33) and IPF (*n* = 22), and healthy controls (*n* = 8) [26]. Pathogens were commonly grown from BAL fluid of patients with WG and IPF. Specifically, the authors observed pathogen growth (e.g., *Haemophilus influenzae*, *Haemophilus parainfluenzae*, *Streptococcus pneumoniae*, *Moraxella catarrhalis*, *Pseudomonas aeruginosa* and *Proteus mirabilis*) in 8/22 (36%) IPF patients, suggesting that occult bacterial infection may contribute to the development of IPF. Garzoni and colleagues employed ultra-deep 16S rRNA gene sequencing, a culture-independent technique, to characterize the microbiota of the upper (by using oropharyngeal swabs) and lower (by using BAL fluid) respiratory tracts from 18 patients with ILD (five with idiopathic interstitial pneumonia (IIP), six with non-IIP ILD and seven with sarcoidosis), six immunocompromised patients with *Pneumocystis* pneumonia and nine healthy controls [27]. The authors established the presence of a lower airway microbiota, dominated by *Prevotellaceae*, *Streptococcaceae* and *Acidaminococcaceae*, but did not observe any significant between-group differences in the composition of the airway microbiota.

The correlating outcomes with biochemical markers to estimate time-progression (COMET)-IPF study was the first to truly evaluate the lung microbiome in patients with moderately severe IPF (*n* = 55) (mean forced vital capacity (FVC) 70.1% and mean diffusing capacity of the lung for carbon monoxide (DL_CO_) 42.3%), although the lack of a control group represents an important weakness of the study [28]. The most commonly identified bacteria were *Prevotella*, *Veillonella* and *Escherichia* spp., all well-known inhabitants of the healthy respiratory microbiome. Moreover, the presence of a specific *Streptococcus* sp. (operational taxonomic units (OUT) 1345) or *Staphylococcus* sp. (OUT 1348) was strongly associated with disease progression, defined as a composite of death, acute exacerbation, lung transplant or relative decline in FVC ≥ 10% or DL_CO_ ≥ 15% over 48 weeks. Over the study period, 36/55 (65%) patients experienced disease progression. Notably, patients predominantly had either *Streptococcus* OTU 1345 or *Staphylococcus* OTU 1348 but not both. However, the enrichment of *Streptococcus* OTU 1345 and *Staphylococcus* OTU 1348 was found in less than half of the patients, implying that these organisms alone cannot fully explain disease pathogenesis or progression. Molyneaux and colleagues prospectively explored the lung microbiomes of patients with IPF, drawing a comparison between healthy and disease (COPD) controls [29]. They demonstrated a statistically significant, two-fold higher bacterial burden in the BAL of patients with IPF (*n* = 65) compared to patients with COPD (*n* = 17) or healthy controls (*n* = 27). Furthermore, and perhaps more interestingly, bacterial burden at baseline predicted the rate of functional decline and risk of death. Notably, the bacterial burden was lower in IPF patients who carried the mutant *MUC5B* rs35705950 T allele, a known risk factor for IPF [8], yet a predictor of longer survival in patients with established disease [30]. Theoretically, this observation would suggest the existence of (at least) two distinct pathogenetic pathways leading to alveolar injury in IPF: one involving bacterial overload in non-carriers of the *MUC5B* rs35705950 mutant variant and another in which carrying the *MUC5B* rs35705950 T allele and *MUC5B* overexpression at the distal airway/alveolar junction results in increased local exposure or aberrant cellular responses to bacterial stimuli [31]. As well as an increased bacterial burden, the authors identified increased reads of potentially pathogenic *Haemophilus*, *Streptococcus*, *Neisseria* and *Veillonella* spp. in IPF patients compared to healthy controls. They were unable to identify any bacterial community structure or composition, which differed between IPF patients with stable or progressive disease.

The intriguing association between clinical outcome and bacterial burden has now been independently validated by the COMET authors. O’Dwyer and colleagues employed digital droplet PCR, a more sensitive measure than previous studies, and clearly replicated the finding that subjects with progressive disease have a higher bacterial burden at the time of diagnosis. The observation that this finding now holds true in independent cohorts using multiple bacterial quantification platforms strengthens the association with bacterial burden and survival in IPF. In this study, changes in bacterial burden were also associated with a reduction of microbial diversity, often associated with disease, and a proinflammatory and profibrotic signal in the airways.

In further work, the COMET authors performed a sub-analysis comparing the microbiota in IPF BAL fluid (*n* = 68) with IPF radiographic features (e.g., presence or absence of honeycombing, a cardinal radiological and histopathologic feature of IPF) [32]. Dickson and colleagues found no association between bacterial burden and the presence of radiological honeycombing. They identified slight differences in the overall bacterial community structure based on radiological change, but no clear candidate bacteria driving these differences were identified. Taken together, these observations suggest that lung microbial communities in patients with IPF differ significantly based on disease morphology and severity, but further prospective work on the geographical variation of the lung microbiome in IPF is needed [32].

Bacteria have the potential to cause epithelial cell injuries in the airways, directly or indirectly, either by inducing a host immune response or by activating the wound healing cascade following a chronic, low-level antigenic stimulus [33]. However, despite growing evidence of association, the causal significance of an altered lung microbiota in IPF remains elusive. Integrated analysis of the host transcriptome and microbial signatures have demonstrated an apparent host response to the presence of an altered or more abundant microbiome, suggesting that the bacterial communities of the lower airways may act as persistent stimuli for repetitive alveolar injury in IPF [34]. In addition, host-microbiome interactions have been shown to influence fibroblast responsiveness and progression-free survival [35], although these, again, remain correlative rather than causal relationships. Recently, O’Dwyer and colleagues validated the finding that lung bacterial burden predicts disease progression in patients with IPF while the diversity and composition of microbiota correlate with increased alveolar profibrotic cytokines [36]. In an effort to understand the mechanisms behind these changes, the authors then employed a mouse model of pulmonary fibrosis. With little known of the effect of bleomycin-induced fibrosis on the respiratory microbiome in mouse models, the authors first set out to characterize this. While no change in bacterial burden was observed, the bacterial diversity increased rapidly during the inflammatory phase of the model and persisted once the development of fibrosis occurred. This suggests that lung dysbiosis precedes the development of fibrosis in these mouse models. To study the impact of the microbiome in this model, the authors then employed germ-free conditions. In sterile conditions, bleomycin exposure resulted in similar levels of fibrosis, but the absence of a microbiota protected against mortality [36]. The limitations of the animal models of IPF notwithstanding, this study provides a potential mechanistic link between lung bacterial burden and disease pathogenesis and progression in pulmonary fibrosis. In contrast with the aforementioned studies suggesting an abnormal bacterial burden and composition in the lung of patients with IPF compared to diseased and healthy controls, Kitsios and colleagues found a surprisingly low bacterial signal in the subpleural lower lobes of lung explants from end-stage patients with IPF [37]. Notably, the low signals in IPF patients were similar to those of negative controls and in striking contrast with the abundance of pathogens identified in cystic fibrosis lung explants. Bias regarding the end-stage population used and/or the analysis of the subpleural honeycomb region where bacterial load could be lower may account for such conflicting results.

## 3. The Role of Infection and Microbial Dysbiosis in Acute Exacerbation of IPF

Acute exacerbations of IPF (AE-IPF) are episodes of acute respiratory worsening with a median survival following the event of approximately three to four months [38]. According to the recently revised definition and diagnostic criteria, they can be either idiopathic or triggered (for instance, by infection), but cardiac failure, fluid overload or extra-parenchymal causes, such as pulmonary embolism, pneumothorax or pleural effusion, need to be excluded [39]. Notably, because the original diagnostic criteria for AE-IPF required these events to be idiopathic [40], studies published before the 2016 revised document have been conducted in patients without overt clinical infection.

Several studies have reported an association between subclinical or occult viral infection and AE-IPF, although the causal role of this association remains to be proven. A study of 43 subjects with AE-IPF failed to clearly identify a viral or other infectious aetiology for the acute event in the vast majority of patients [41]. In addition, all subjects (*n* = 43) had negative bacterial cultures and negative viral serology. By PCR analysis of BAL fluid, 4/43 patients tested positive for common respiratory viruses (e.g., parainfluenza (*n* = 1), rhinovirus (*n* = 2) and coronavirus (*n* = 1)), while no viruses were detected in the BAL fluid from stable patients (*n* = 40). Pan-viral microarrays revealed the presence of HSV (*n* = 1), EBV (*n* = 2) and Torque Teno virus (TTV) virus (*n* = 12) in patients with AE but not in the stable disease group (*p* = 0.0003), but TTV infection was present in a similar percentage of diseased controls with acute lung injury. Deep sequencing of a subset of AE cases confirmed the presence of TTV but did not identify additional viruses [41]. A Japanese study of 78 patients with AE of ILD, including 27 with IPF, found viruses in the respiratory samples of 15 of them (19.2%), including HHV7 (*n* = 4) and HHV7 plus CMV (*n* = 3), but the proportions of virus infections in the IPF and non-IPF ILD groups were similar [42]. Moreover, while the probability of survival over 60 days was lower in the virus positive group, virus isolation itself did not predict 60-day survival, questioning the clinical relevance of these findings. More recently, viral sequences were detected in the nasopharyngeal swab of 18/30 (60%) patients with AE-IPF and 13/30 (43.3%) cases with stable disease (*p* = 0.2). AE-IPF showed increased levels of the inflammatory cytokines IL-6, IFN-gamma, MIG, IL-17 and IL-9 compared to IPF patients with stable disease and controls. HHV and Influenza virus A accounted for the majority of the viral burden [43]. Interestingly, AE-IPF following influenza A vaccination has been reported [44].

Until recently, there has been little focus on the role of bacterial infection as the trigger of AE-IPF.

Molyneaux and colleagues used culture-independent techniques to explore changes in the BAL microbiota from patients with stable IPF (*n* = 15) and subjects experiencing AE-IPF (*n* = 20) [45]. Despite negative BAL bacterial cultures and virus screens, the bacterial burden of patients with AE-IPF was over four times higher than that of patients with stable disease. In addition, while the bacterial community of patients with stable disease contained *Streptococcus*, *Prevotella*, *Veillonella*, *Haemophilus* and *Psedomonas*, following AE-IPF the microbiota changed substantially, with an increase in *Campylobacter* and *Stenotrophomonas* spp. and a decrease in *Veillonella* sp. [45]. More recently, Weng and colleagues looked at the presence of pathogens and specific IgM against microbial pathogens in sputum, and sequences of pathogens in nasopharyngeal swabs from 170 IPF patients (122 with stable disease and 48 with AE-IPF) and 70 controls [43]. Bacterial IgM was higher in stable IPF than in controls and in AE-IPF than in stable patients, with *Mycoplasma* displaying the highest IgM positive rate in both disease subsets (12.2% and 5.6%, respectively). Thirty-eight different bacterial strains (mainly Gram-negative) were detected in the sputum of patients with IPF but the total detection rates did not differ between patients with AE-IPF and those with stable disease (18.8% versus 21.3%, respectively).

Taken together, these observations suggest that alterations in pulmonary microbiome play a causative role in at least some cases of AE-IPF. However, theoretically, the higher bacterial load and altered microbiome found during an AE could be the consequence (rather than the cause) of the diffuse alveolar damage characteristic of AE. Ideally, future studies should collect paired samples from the same patients when stable and during the acute event to prove any changes from the baseline microbiota, forcing the relationship from association to causation [46].

## 4. Modulation of Lung Microbiome as a Novel Therapeutic Option

Despite the rationale for their use in IPF, very few studies have investigated the efficacy of antibiotics in this setting. Following a pilot study of 20 patients with progressive fibrotic lung disease in which co-trimoxazole treatment improved FVC; shuttle walk distance with reduced oxygen desaturation during exercise; the Medical Research Council (MRC) dyspnoea score; and St. George’s Respiratory Questionnaire (SGHRQ) symptom score [47], a larger, randomized, double-blind, placebo-controlled trial was designed to assess the safety and efficacy of oral co-trimoxazole 960 mg twice daily for 12 months in addition to usual treatment in patients with fibrotic IIP (definite or probable IPF, *n* = 170; definite or probable nonspecific interstitial pneumonia (NSIP), *n* = 11) [48]. No significant between-group differences were seen with regard to change in FVC, the primary outcome. Similarly, no difference between the co-trimoxazole and placebo groups was observed in terms of change in DL_CO_, 6-min walk test (6MWT), or MRC dyspnoea score in the intention-to-treat analysis. However, in the per-protocol analysis, co-trimoxazole treatment compared with placebo was associated with a significant improvement in EuroQol (EQ5D)-based utility (a measure of health state), a significant reduction in the percentage of patients requiring an increase in oxygen therapy and a significant reduction in all-cause mortality (3/53 deaths in the co-trimoxazole group versus 14/65 in the placebo group; *p* = 0.02, hazard ratio [HR] 0.21) [48]. The authors speculated that the reduced mortality in the co-trimoxazole group could be due to a reduction in the rate of respiratory infection. In fact, patients receiving “as usual treatment,” immunosuppressive treatment—which at the time of the study was still a therapeutic option in patients with IPF—were more likely to die if they were in the placebo group, whereas baseline immunosuppressive therapy did not have an effect on mortality in the co-trimoxazole group. The study, however, had important drawbacks, including the lack of a true placebo arm and the high dropout rate due to side effects (mostly rash and nausea) among patients receiving co-trimoxazole (28/92, 30% versus 7/86, 8% in the placebo group).

A phase III double blind, randomised, placebo-controlled, multi-centre clinical trial of oral co-trimoxazole versus placebo in 330 patients with moderate and severe IPF is currently underway (EME-TIPAC—The Efficacy and Mechanism Evaluation of Treating Idiopathic Pulmonary Fibrosis with the Addition of Co-trimoxazole; EudraCT number 2014-004058-32) [49]. The primary outcome is the time to death (all causes), lung transplant or the first non-elective hospital admission.

The potential benefit of macrolides has also been evaluated in IPF patients, although only in retrospective, relatively small cohorts. In a single-centre study, Kawamura and colleagues compared the efficacy of azithromycin 500 mg/day for five days with that of a fluoroquinolone-based regimen from a historical control group in 85 patients with idiopathic AE-IPF [50]. Mortality was significantly lower among patients treated with azithromycin (*n* = 38) than in those treated with fluoroquinolones (*n* = 47; 26% versus 70%; *p* < 0.001, HR 0.28). Multivariate analysis confirmed that azithromycin use was independently correlated with 60-day mortality. More recently, in a retrospective observational study, Macaluso and co-workers looked at 108 IPF patients receiving prophylactic azithromycin (250 mg three times weekly) to evaluate its long-term effect on the rate of hospitalization. To this end, they compared the number of all-cause non-elective hospitalizations and antibiotic courses in the 12 months preceding and following treatment initiation, and found that both hospital admissions (seven versus 31) and antibiotic courses (40 versus 176) were significantly lower in the year after institution of prophylactic azithromycin [51]. Overall, azithromycin was safe and well tolerated. These data suggest a potential clinical benefit of prophylactic azithromycin in patients with IPF. However, the mechanism/s (antibacterial or anti-inflammatory) by which macrolides reduce the risk of hospitalization events in patients with IPF remain to be elucidated.

Table 1 summarizes currently ongoing clinical trials evaluating the efficacy and safety of antibiotics in IPF.

## 5. Looking beyond the Lung: The Potential Role of the Gut Microbiome and Gut–Lung Axis in the Pathogenesis of Pulmonary Fibrosis

**Figure 1:**
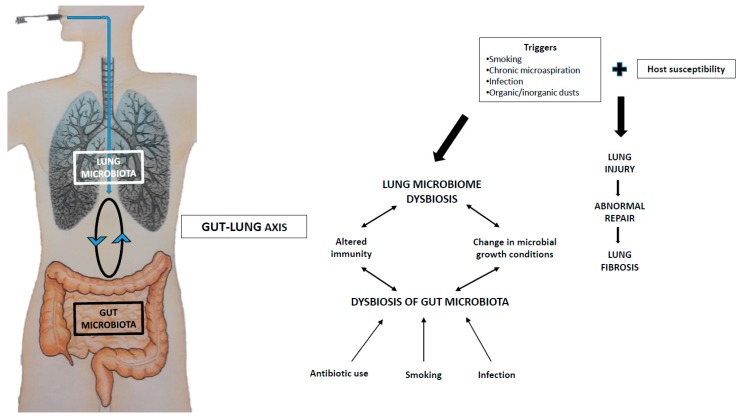
Hypothetical model of host–microbiota interaction in idiopathic pulmonary fibrosis (IPF): the gut–lung axis. Bacteria have the ability to modulate local (e.g., lung and gut) and systemic immunity. When the gut microbiota is altered, for example, during infection or antibiotic use, the microbiota-derived signals are altered too, leading to changes in the immune response against pathogens. In the lung, smoking, organic/inorganic dusts, infection and the chronic microaspiration of gastric content, among other things, modulates the composition of the microbiota, which, in turn, induces an altered immune response against pathogens. The existence of a gut–lung axis perpetuates this vicious circle. In this scenario, specific microbiota strains (e.g., probiotics), which have proven successful in the treatment of several intestinal disorders, may also benefit fibrotic lung disease by restoring the integrity and efficiency of the lung microbiome.

A balanced intestinal microbial community is essential for the development and maintenance of immune function and health. The respiratory microbiome develops in tandem with that of the gut [52]. There is clear cross-talk between the two compartments (the “gut–lung axis”) and manipulation of the gut microbiome, by changes in diet or drugs, can alter the microbiome of the lung, providing beneficial effects in asthma [53] and protection against respiratory viruses [54]. Direct seeding of bacteria from the gastrointestinal tract into the airways may play a role in shaping the respiratory microbiome and triggering local immune responses (Figure 1). However, it is now clear that the gastrointestinal tract can direct immune responses in remote environments by the systemic dissemination of bacterial metabolites via the bloodstream, as has been shown for short-chain fatty acids (SCFAs). The beneficial effects of dietary-fibre fermentation products are now well documented in a number of chronic inflammatory diseases [55]. Dysbiosis of the gut microbiota has been associated with a number of local and systemic conditions, including respiratory diseases. It is the case of asthma, wherein the gut microbiota of patients is enriched for histamine-producing bacteria compared to healthy controls [56]. Dysbiotic gut microbiota has also been shown in patients with systemic sclerosis, particularly those with extra-intestinal manifestations, including lung fibrosis [57] and silicosis [58]. In experimental systemic sclerosis, manipulation of intestinal microbiota through early-life antibiotic administration was associated with dysregulated T-cell responses in the lung and altered expression of fibrosis-related genes [59]. Moreover, early-life dysbiosis was associated with adult-onset lung fibrosis. The hypothesis that early-life intestinal dysbiosis is durable and confers susceptibility to late-onset lung fibrosis in human disease is intriguing. However, while the role of the gut–lung axis in pulmonary inflammation has been studied, little is known about the impact of microbial metabolites on the development of pulmonary fibrosis.

## 6. Conclusions

Several studies have suggested that altered microbiome burden, diversity and composition may contribute to disease pathogenesis, progression, acute exacerbation and mortality in IPF. Should a mechanistic link between a deranged lung microbiome and IPF development and progression be established, microbiome manipulation, with the aim of restoring a “healthy” microbiome community, will soon represent a potential therapeutic intervention for IPF, although a holistic approach to account for the many factors driving disease development, progression and acute worsening is more likely to be truly efficacious. Whether the lung microbiome should be manipulated by using antibiotics, probiotics (extrinsic microbes administered in the interests of health) or prebiotics (molecules that promote specific bacterial growth) is unknown. Regardless, microbiome manipulation should target pathogenic microbes without altering the residential members of the microbial community [60]. That would be even more challenging.

## Figures and Tables

**Table 1:** Clinical trials evaluating the efficacy and safety of antibiotics in idiopathic pulmonary fibrosis.

Study Name	Study Design	Study Duration	Status	Primary Outcome	Estimated Enrolment/Inclusion Criteria	Trial Number
Azithromycin for the Treatment of Cough in Idiopathic Pulmonary Fibrosis—A Clinical Trial	Single centre, prospective, double blind, randomized, 2 treatments, 2 period cross-overPlacebo versus Azithromycin 500 mg/d three times weekly	Two 12-week treatment periods separated by a 4-week drug-free washout period	Completed	Subjective response to treatment (1.3 unit reduction of cough as measured with Leicester Cough Score)	25 patientsAge ≥ 18 years, IPF diagnosis, symptoms of cough	NCT02173145
Study of Clinical Efficacy of Antimicrobial Therapy Strategy Using Pragmatic Design in Idiopathic Pulmonary Fibrosis (cleanUp-IPF)	Phase III, randomized, un-blinded, multi-centreTrimethoprim/Sulfamethoxazole (T/S) 160/800 mg twice daily OR doxycicline 100 mg/d if T/S is not indicated	42 months	Recruiting	Time to first non-elective respiratory hospitalization or all-cause mortality	500 patientsAge ≥ 40 years, IPF diagnosis	NCT02759120
The Efficacy and Mechanism Evaluation of Treating Idiopathic Pulmonary Fibrosis with the Addition of Co-Trimoxazole (EME-TIPAC)	Phase III, double blind, parallel group, randomized, placebo controlled multicentreCo-trimoxazole 960 mg twice daily versus placebo	Between 12 and 42 (median 27) months	Recruiting	Time to death (all causes), lung transplant or the first non-elective hospital admission	330 patientsAge > 40 years, MRC dyspnoea score > 1, on stable treatment regimen for at least 4 weeks *, IPF diagnosis	EUDRACT 2014-004058-32

* Oral prednisolone up to 10 mg/d, anti-oxidant therapy, Pirfenidone, Nintedanib or other lensed medication for IPF.
